# Animal models of allergic rhinitis: current progress and advances in traditional Chinese medicine syndrome models

**DOI:** 10.3389/falgy.2026.1894329

**Published:** 2026-08-12

**Authors:** Chuwen Yang, Shu Shuen Yee, Haifeng Feng, Jun Zhao

**Affiliations:** Department of Pediatrics, Shuguang Hospital Affiliated to Shanghai University of Traditional Chinese Medicine, Shanghai, China

**Keywords:** allergic rhinitis, animal models, disease-syndrome integration, model evaluation, traditional Chinese medicine syndromes

## Abstract

**Background:**

Allergic rhinitis (AR) is a common chronic inflammatory disease of the nasal mucosa, with a rising global prevalence. Traditional Chinese Medicine (TCM) has demonstrated therapeutic efficacy in AR, yet the mechanistic understanding of TCM interventions remains incomplete. The development of AR animal models that integrate TCM syndrome characteristics—referred to as disease-syndrome combination models—represents a crucial bridge between TCM theory and clinical application. This systematic review aims to summarize the current landscape of animal models of allergic rhinitis (AR), with particular emphasis on disease–syndrome integrated models based on Traditional Chinese Medicine (TCM) syndromes.

**Methods:**

We systematically searched the China National Knowledge Infrastructure (CNKI), VIP Database, Wanfang Database, and PubMed for studies on AR animal models and TCM syndrome models published between January 1995 and May 2026. Data were extracted and analyzed regarding animal strains, sensitization protocols, TCM syndrome induction methods, immunological parameters, and model evaluation criteria. The review was conducted in accordance with the PRISMA 2020 guidelines and structured using the PECO framework.

**Results:**

Among the 470 included studies, 421 investigated conventional AR animal models, whereas 49 established disease–syndrome integrated models incorporating TCM syndrome characteristics. The most commonly used animal models were SD rats (37.02%) and BALB/c mice (37.65%). Ovalbumin (OVA) was the predominant sensitizing allergen (92.97%), while Lung Qi Deficiency (cold) was the most frequently modeled TCM syndrome (34.69%). Syndrome induction methods included tobacco smoke exposure, cold stimulation, bitter-cold herbal gavage, and physical exhaustion. Distinct immunological profiles were observed across syndromes: the Lung Qi Deficiency-Cold pattern was characterized by Th1/Th2 imbalance; the Spleen Qi Deficiency pattern involved gut microbiota dysbiosis and Treg/Th17 imbalance; and the Kidney Yang Deficiency pattern exhibited neuro-endocrine-immune axis suppression. Model evaluation integrated behavioral scoring, immunological assays, histopathological examination, and TCM syndrome-specific phenotyping.

**Conclusions:**

Current evidence is dominated by conventional AR animal models, whereas disease–syndrome integrated TCM models represent a relatively small but rapidly expanding subset of the literature. Standardization of naturally relevant allergen models, development of objective syndrome-reflective biomarkers, and establishment of a “symptom-immunology-omics” multidimensional evaluation system are urgently needed to enhance the reliability and translational value of TCM syndrome animal models in AR research.

**Systematic Review Registration:**

PROSPERO: CRD420261466344.

## 1 Introduction

Allergic rhinitis (AR) is one of the most common chronic inflammatory diseases of the upper airway, characterized by paroxysmal sneezing, rhinorrhea, nasal itching, and nasal congestion. With accelerating industrialization, ecological changes, and shifting lifestyles, the global prevalence of AR has risen markedly over recent decades (1). Epidemiological surveys estimate that AR affects approximately 10%–30% of the global population, with a particularly pronounced increase among children and adolescents. Beyond its direct nasal symptoms and associated sleep disturbances, AR substantially impairs learning efficiency, work productivity, and overall quality of life, positioning it as a significant public health burden worldwide (2).

Animal models have become indispensable tools for investigating the immunopathogenesis of AR and for evaluating potential therapeutic interventions. Over the past three decades, a variety of animal species—including rats, mice, guinea pigs, rabbits, and sheep—have been employed using different sensitizing allergens such as ovalbumin (OVA), house dust mite (HDM), pollen, and toluene-2,4-diisocyanate (TDI). These models differ substantially in their immunological characteristics, clinical relevance, reproducibility, and suitability for mechanistic or pharmacological studies. Consequently, selecting appropriate animal species, allergens, adjuvants, and evaluation methods has become a critical issue in experimental AR research.

Traditional Chinese Medicine (TCM) has a long history of treating AR with proven clinical efficacy, and mechanistic studies have advanced to the cellular and molecular levels. Consequently, elucidating the mechanisms of TCM interventions and establishing robust disease-syndrome integrated animal models that recapitulate both AR pathology and TCM syndrome characteristics have become essential translational tools bridging TCM theory and clinical practice (3, 4). Since Wang et al. first constructed a combined AR model with Wei Qi deficiency in 1995 based on Tanaka's AR disease model, significant progress has been made in this field in China (5, 6).

Despite these advances, current evidence remains heavily weighted toward conventional AR animal models. Disease–syndrome integrated TCM models constitute only a relatively small proportion of the published literature and lack standardized protocols for syndrome induction, objective syndrome-specific biomarkers, and unified evaluation criteria.

This review systematically summarizes the current evidence on animal models of allergic rhinitis, with particular emphasis on disease–syndrome integrated TCM models,focusing on animal selection, allergen and adjuvant choices, sensitization protocols, syndrome induction strategies, mechanistic investigations, and evaluation criteria. Our goal is to provide a comprehensive reference for improving model standardization, enhancing translational relevance, and promoting future mechanistic and preclinical research on allergic rhinitis.

## 2 Methods

### 2.1 Study design and reporting standards

This systematic review was conducted in accordance with the Preferred Reporting Items for Systematic Reviews and Meta-Analyses 2020 (PRISMA 2020) guidelines to enhance transparency, reproducibility, and methodological rigor. The study question was structured using the PECO framework (Population, Exposure, Comparator, Outcome) to explicitly define the scope of animal model research.

### 2.2 PECO framework for research questions

#### 2.2.1 Population

Experimental animals, including rats, mice, guinea pigs, rabbits, and sheep, used to establish AR models or AR TCM syndrome models.

#### 2.2.2 Exposure

AR modeling methods [sensitization/challenge with ovalbumin [OVA], house dust mite [HDM], pollen, or toluene-2,4-diisocyanate [TDI]] and TCM syndrome induction methods (Lung Qi Deficiency, Spleen Qi Deficiency, Kidney Yang Deficiency, Lung Heat, and Cold-Heat Complex).

#### 2.2.3 Comparator

Comparisons between different AR modeling approaches, different TCM syndrome induction methods, or disease models vs. disease-syndrome combined models.

#### 2.2.4 Outcome

Model success and evaluation metrics, including behavioral scores (sneezing, nasal rubbing), histopathological changes (nasal mucosal inflammation, eosinophil infiltration, goblet cell hyperplasia), immunological parameters (IgE, OVA-sIgE, IL-4, IL-5, IL-13, IFN-γ), and TCM syndrome-specific indicators (body weight, activity, fur condition, food/water intake, defecation).

#### 2.2.5 Study design

Animal experiments, including RCT studies and observational studies.

### 2.3 Strategy

#### 2.3.1 Databases

We systematically searched PubMed, CNKI, VIP, and Wanfang databases for studies published between January 1, 1995, and May 1, 2026. Web of Science was explored during the preliminary scoping stage of this review. However, because a large proportion of records retrieved from Web of Science overlapped with those identified in PubMed and Chinese databases, and because the current review focused specifically on animal experiments and TCM syndrome-integrated AR models, the final formal systematic search was restricted to PubMed, CNKI, Wanfang, and VIP, which were considered the most relevant sources for the predefined review question. This decision was made to optimize the balance between comprehensiveness and review feasibility, as we judged that the four selected databases adequately covered the core literature in both English and Chinese. This decision was made before the final data extraction stage and is now explicitly reported for transparency.

The search strategies combined MeSH terms and free-text keywords without language restrictions. Boolean operators (AND/OR) were applied according to each database's search rules. Tables 1, 2 present the detailed search strategies for PubMed and Chinese databases, respectively. No filters for study design, publication type, or language were applied during the electronic search to maximize sensitivity.

**Table 1:** Search strategy for the PubMed database.

No.	Search terms
#1	MeSH terms: “Rhinitis, Allergic”
#2	Title/Abstract: “allergic rhinitis” OR “allergic nasal inflammation” OR “allergic rhinopathy”
#3	#1 OR #2
#4	MeSH terms: “Models, Animal” OR “Disease Models Animal”
#5	Title/Abstract: “animal model” OR “experimental model” OR “allergic rhinitis model” OR “AR model”
#6	#4 OR #5
#7	MeSH terms: “Rats” OR “Mice” OR “Guinea Pigs” OR “Rabbits”
#8	Title/Abstract: rat OR rats OR mouse OR mice OR murine OR guinea pig OR rabbit OR rodent
#9	#7 OR #8
#10	Title/Abstract: “traditional Chinese medicine syndrome” OR “TCM syndrome” OR “syndrome model” OR “lung qi deficiency” OR “lung deficiency” OR “spleen deficiency” OR “kidney yang deficiency” OR “lung heat” OR “heat syndrome” OR “cold-heat complex” OR “cold and heat in complexity”
#11	#3 AND #6 AND #9
#12	#3 AND #6 AND #10
#13	#11 OR #12

**Table 2:** Example search strategy for Chinese databases (CNKI/VIP/wanfang).

No.	Search terms
#1	过敏性鼻炎(Allergic Rhinitis)
#2	动物模型 OR 实验研究(Animal models OR experimental studies)
#3	大鼠 OR 小鼠 OR 豚鼠 OR 兔(Rats OR Mice OR Guinea Pigs OR Rabbits)
#4	中医证候 OR 肺气虚 OR 脾虚 OR 肾阳虚 OR 肺热 OR 寒热错杂(Traditional Chinese Medicine Syndromes OR Lung Qi Deficiency OR Spleen Deficiency OR Kidney Yang Deficiency OR Lung Heat OR Mixed Cold and Heat)
#5	#1 AND #2 AND #3
#6	#1 AND #2 AND #4
#7	#5 OR #6

### 2.4 Inclusion and exclusion criteria

#### 2.4.1 Inclusion criteria

Studies were included if they met all of the following: (1) conventional AR animal models, including model construction, optimization, or evaluation; (2) disease–syndrome integrated AR models incorporating TCM syndrome characteristics; (3) full-text availability; (4) sufficient reporting to extract at least animal species, modeling method, or primary outcome data.

#### 2.4.2 Exclusion criteria

Studies were excluded if they met any of the following: (1) reviews, editorials, systematic reviews, meta-analyses, or progress reports; (2) conference abstracts, meeting papers, or study protocols not constituting original research; (3) non-animal studies (clinical research, cell culture, or *in vitro* experiments); (4) studies not addressing AR or using non-AR models Or experimental studies on AR comorbidities; (5) duplicate publications; (6) studies for which the full text could not be retrieved; (7) Animal experiments involving allergic rhinitis comorbidity. (8) studies with critically insufficient methodological or outcome information, such that essential data on animal species, modeling protocol, syndrome induction, or outcome assessment could not be extracted reliably.

### 2.5 Study selection process

All retrieved records were imported into reference management software, and duplicates were removed. Two independent reviewers performed title/abstract screening and full-text assessment according to the predefined eligibility criteria. Disagreements were resolved through discussion with a third reviewer. The screening process was documented following the PRISMA 2020 flow diagram to ensure transparency and traceability.

### 2.6 Data extraction

Two reviewers independently extracted data from the included studies and cross-checked for accuracy. Extracted items included: (1) basic bibliographic information (first author, publication year, journal); (2) animal characteristics (species, strain, sex, age, body weight); (3) AR modeling parameters (allergen type, adjuvant, sensitization route, challenge method, dosage, modeling duration); (4) TCM syndrome modeling parameters (syndrome type, induction method, duration/frequency); (5) outcome measures (behavioral, histopathological, immunological, and syndrome-specific indicators); and (6) methodological characteristics (randomization, blinding, sample size estimation, attrition, and selective reporting).

### 2.7 Assessment of methodological quality

Given that all included studies were animal experiments and many lacked complete reporting of experimental design and implementation, we did not apply a formal risk-of-bias tool such as SYRCLE. Instead, we performed a descriptive evaluation of key methodological quality elements across five domains: (1) randomization—whether random group allocation was explicitly reported; (2) blinding—whether blinding was applied during modeling, outcome assessment, or histopathological scoring; (3) sample size calculation—whether sample size estimation or justification was reported; (4) incomplete outcome data—whether animal deaths, attrition, or exclusions were reported with reasons; and (5) selective reporting—whether outcomes listed in Methods were fully reported in Results. Two reviewers independently extracted these data, with disagreements resolved by a third reviewer. Descriptive statistics were used to summarize the reporting frequency of each methodological item.

## 3 Results

### 3.1 Literature search and screening results

**Figure 1:**
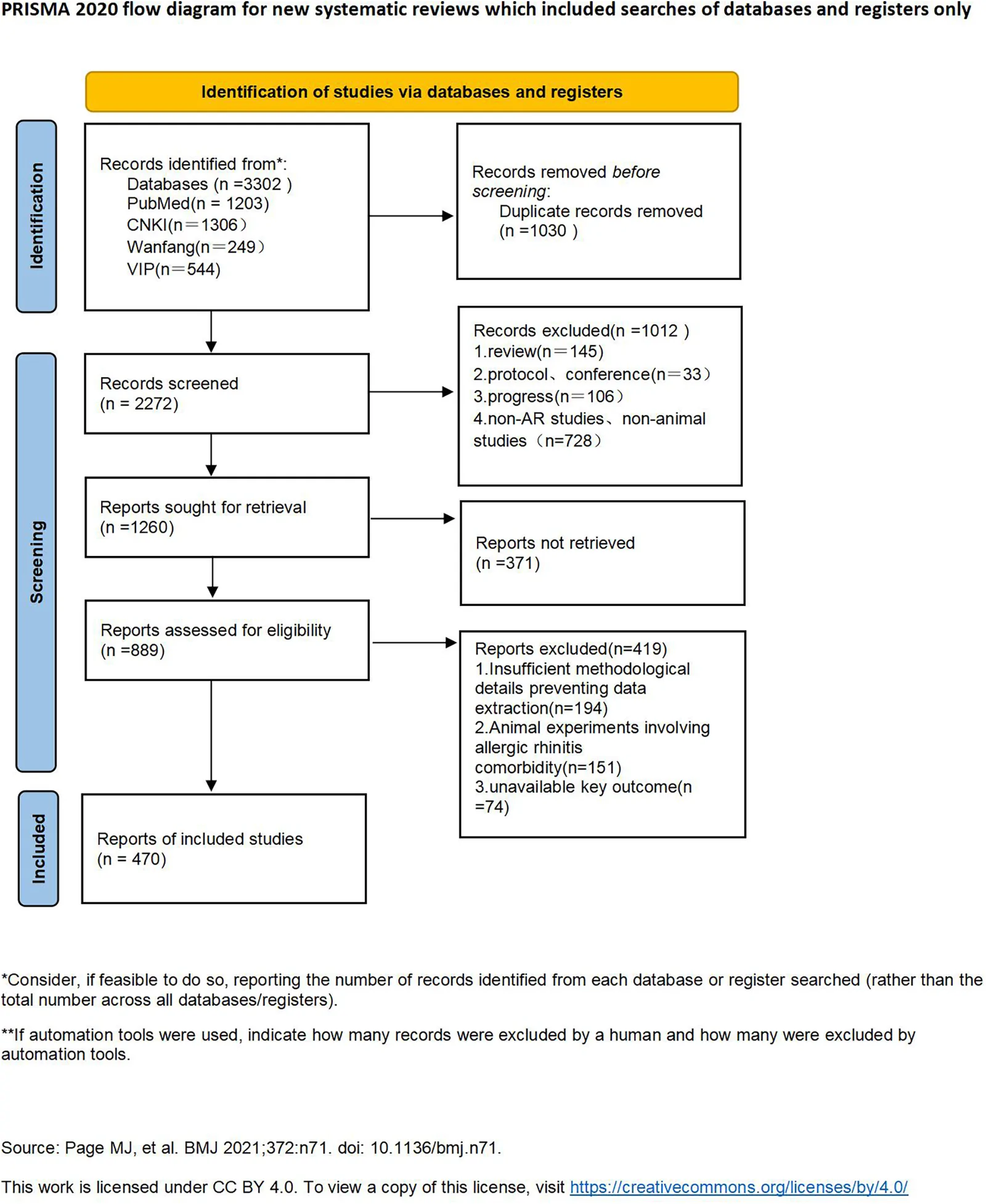
PRISMA 2020 flow diagram illustrating the study selection process for this systematic review. Records were retrieved from PubMed, CNKI, Wanfang and VIP databases. The diagram presents the number of records identified, screened, assessed for eligibility, and finally included, alongside detailed reasons for exclusion at each stage.

The systematic search yielded 3,302 records across all databases (PubMed: 1,203; CNKI: 1,306; Wanfang: 249; VIP: 544). After removing 1,030 duplicates, 2,272 records underwent title/abstract screening, during which 1,012 were excluded (145 reviews, 33 protocols/conference papers, 106 progress reports, 728 non-AR or non-animal studies). Of the remaining 1,260 records sought for full-text retrieval, 371 were unavailable, leaving 889 articles for full-text eligibility assessment. Following full-text review, 419 studies were excluded because they did not meet the predefined eligibility criteria, most commonly due to insufficient methodological or extractable modeling information. 470 studies were included in the final review. The screening process is illustrated in the PRISMA flow diagram (Figure 1).

### 3.2 Methodological quality results

A summary of the methodological quality assessment is presented in Table 3. Overall, the reporting quality of the included studies was suboptimal. Baseline animal characteristics—including species, strain, sex, age, and body weight—were adequately described in all 470 studies (100.00%). Although all studies reported that animals were randomly allocated to groups, only 160 (34.04%) specified the method used to generate the random sequence. Blinding was reported in merely 2 studies (0.42%), and sample size calculation or justification was provided in only a small proportion of studies (92/470, 19.57%). Attrition or exclusion of animals, along with the corresponding reasons, was documented in just 20 studies (4.25%). Collectively, these findings indicate that methodological transparency in AR animal model studies remains limited, which may compromise internal validity, hamper cross-study comparability, and undermine the reproducibility of experimental findings.

**Table 3:** Methodological quality reporting of the included studies (*n* = 470).

Item	Definition	*n* (%)
Random allocation reported	Study explicitly stated animals were randomly assigned to groups	470 (100.00)
Method of random sequence generation reported	Random number table/computer-generated randomization etc. described	160 (34.04)
Blinding reported	Blinding of modeling, outcome assessment, or histopathology scoring described	2 (0.42)
Sample size calculation reported	*A priori* sample size estimation or justification provided	92 (19.57)
Incomplete outcome data addressed	Attrition/deaths/exclusions reported with reasons	20 (4.25)
Selective reporting suspected	Outcomes described in Methods but incompletely reported in Results	0 (0.00)
Baseline animal characteristics reported	Species/strain/sex/age/weight adequately described	470 (100.00)

### 3.3 General characteristics of included studies

Among the 470 included studies, 421 (89.6%) investigated conventional AR animal models, whereas 49 (10.4%) established disease–syndrome integrated models incorporating TCM syndrome characteristics. A summary of key characteristics of all included studies and disease–syndrome integrated models incorporating TCM syndrome is provided in Table 4. Supplementary Table S4. provides the complete data. The general characteristics reported below refer to all included studies unless otherwise specified, whereas analyses of TCM syndrome subtypes are restricted to the predefined subgroup of disease–syndrome integrated models (*n* = 49).

**Table 4:** Characteristics of the included studies (*n* = 470), with disease–syndrome integrated TCM models analyzed as a predefined subgroup.

Characteristic	Category	*n* (%)
Study category	Conventional AR animal models	421 (89.57)
	Disease–syndrome integrated TCM	49 (10.43)
Species	Rat	225 (47.87)
	Mouse	226 (48.08)
	Guinea pig	13 (2.76)
	Rabbit	6 (1.27)
	Dogs	1 (0.21)
	goat	1 (0.21)
Rat strain	SD Rats	174 (37.02)
	Wistar rat	51 (10.85)
Mouse strain	BALB/c mouse	177 (37.65)
	C57BL/6 mouse	40 (8.51)
	ICR mouse	4 (0.85)
	Kunming mice	3 (0.63)
	129/sv mouse	2 (0.42)
Sex	Male	209 (44.46)
	Female	156 (33.19)
	Both	105 (22.34)
Allergen	OVA	437 (92.97)
	HDM	17 (3.61)
	Pollen	12 (2.55)
	TDI	5 (1.06)
Adjuvant	Aluminum hydroxide	435 (92.55)
	Pertussis	7 (1.48)
	DPT	2 (0.42)
	Freund adjuvant	3 (0.63)
	None	31 (6.59)
Sensitization route	Intraperitoneal	422 (89.78)
	Subcutaneous	12 (2.55)
	Intranasal	18 (3.82)
	Mixed	13 (2.76)
Challenge route	Intranasal	451 (95.95)
	Aerosol	40 (8.51)
	Other	2 (0.42)
Outcomes	Behavior	358 (76.17)
	Histology	372 (79.14)
	Serology	335 (71.27)
	Molecular Biology	313 (66.59)

Disease–syndrome integrated models (*n* = 49)		
Characteristic	Category	*n* (%)
TCM syndrome subtype	Lung Qi Deficiency (Cold)	17 (34.69)
	Spleen Qi Deficiency	10 (20.40)
	Lung-Spleen Qi Deficiency	7 (14.28)
	Kidney Yang Deficiency	6 (12.24)
	Cold-Heat Complex	3 (6.12)
	Spleen-Kidney Yang Deficiency	2 (4.08)
	Spleen Deficiency with Dampness Retention	2 (4.08)
	Lung-Kidney Yang Deficiency	1 (2.04)
	heat-pattern	1 (2.04)

The most frequently used experimental animals were rats and mice. OVA was the predominant allergen, with aluminum hydroxide, pertussis, and DPT (diphtheria-pertussis-tetanus) being the main adjuvants. The most common TCM syndrome types were Lung Qi Deficiency, Spleen Qi Deficiency, and Kidney Yang Deficiency, induced by cold stimulation, tobacco smoke exposure, exhaustive swimming, fasting, or herbal depletion. Outcome measures included behavioral scores (sneezing, nasal rubbing), histopathological changes (nasal mucosal inflammation, eosinophil infiltration, goblet cell hyperplasia), immunological markers (IgE, OVA-sIgE, IL-4, IL-5, IL-13), and TCM syndrome-specific parameters (body weight, fur condition, food/water intake, activity level).

### 3.4 Selection of model animals

The choice of experimental animals is critical for model success and applicability. Rats, mice, guinea pigs, rabbits, sheep, and dogs have all been used for AR modeling. The most prevalent were SD rats and BALB/c mice. Rats offer stable genetic traits, well-characterized olfactory responses, high sensitivity to environmental dust and irritants, robust IgE antibody production to inhaled allergens, and prominent delayed-type hypersensitivity, making them suitable for AR symptom induction. Male rats are preferred to minimize sex hormone interference and enhance homogeneity (3). Mice, owing to their well-developed lymphatic systems and fully sequenced genomes, provide unique advantages for studying immunological and genetic mechanisms; however, their small size presents technical challenges for blood collection and nasal mucosal sampling (7).

Guinea pigs were among the earliest animals used for AR modeling (8, 9). Their biological characteristics include allergic responses closely resembling those of humans, high serum complement levels, and consistent production of high-titer specific IgE and IgG antibodies. Under identical sensitization conditions, guinea pigs more readily develop pronounced nasal sensitization and are therefore widely used in AR pathogenesis and pharmacotherapy research (7). However, their relative fragility, requirement for strictly controlled environments, strong reactivity to allergens, higher mortality rates, and the scarcity and cost of immunological reagents have led to decreased usage compared to rats and mice (10).

### 3.5 Selection of allergens and adjuvants

#### 3.5.1 Allergens

##### 3.5.1.1 OVA

OVA was the most widely used sensitizing agent, accounting for 92.97% of studies (Table 4). Due to its stable source, high purity, and low batch-to-batch variability, OVA reliably induces IgE-mediated Th2 immune responses when combined with adjuvants such as aluminum hydroxide, making it the classic AR model allergen. However, OVA is not a common human inhaled allergen, and its sensitization requires intraperitoneal injection with exogenous adjuvants, differing from natural inhalation sensitization and limiting clinical relevance. Nevertheless, its high success rate, excellent reproducibility, and well-established evaluation systems make OVA the preferred allergen for pharmacodynamic evaluation and TCM syndrome modeling (11).

##### 3.5.1.2 HDM

HDM is a clinically relevant inhaled allergen containing natural allergens such as Der p1 and Der p2, along with natural adjuvant-like components including lipopolysaccharide (LPS), β-glucan, and chitin. These components can directly activate epithelial cells and dendritic cells, inducing TSLP, IL-25, and IL-33 release and initiating innate Th2 inflammation, thus better simulating natural inhalation sensitization (12).

##### 3.5.1.3 Pollen

Pollen models are primarily used for seasonal AR research and simulate natural pollen exposure-induced nasal inflammation, offering good clinical relevance. However, pollen antigen composition varies regionally, and extraction processes lack standardization, resulting in lower reproducibility compared to OVA and HDM models (13).

##### 3.5.1.4 TDI

TDI is a low-molecular-weight chemical sensitizer that induces neurogenic inflammation and Th2-type inflammatory responses with good reproducibility and was previously widely used. However, due to its inherent toxicity, which can cause animal mortality and endanger personnel, its application has declined (14).

#### 3.5.2 Adjuvants

##### 3.5.2.1 Aluminum hydroxide adjuvant

Aluminum hydroxide is the most commonly used adjuvant in animal experiments. Its mechanisms include: (1) forming a depot for sustained antigen release to stimulate antibody production; (2) inducing inflammation to recruit and activate antigen-presenting cells; and (3) converting soluble antigens into particulate forms for phagocytosis by macrophages, dendritic cells, and B cells. Through “membrane lipid interaction-Syk/PI3K activation” combined with “uric acid/DNA-IRF3 signaling” and “IL-33 release,” it inhibits IL-12 and activates Th2 master transcriptional programs via multiple pathways, efficiently inducing Th2-biased immunity (15, 16).

##### 3.5.2.2 Pertussis adjuvants

Pertussis toxin (PT), used as an adjuvant, is either purified toxin or inactivated Bordetella pertussis. PT increases vascular permeability, inhibits Gi protein-coupled signaling, promotes dendritic cell maturation and IL-12 secretion, and enhances Th1 responses, thereby improving sensitization efficiency to antigens such as OVA (17).

### 3.6 AR modeling methods

#### 3.6.1 OVA-Based modeling

The classical OVA modeling protocol follows a two-phase “systemic sensitization + local challenge” strategy. Base sensitization is typically achieved by intraperitoneal injection of OVA with aluminum hydroxide adjuvant, followed by intranasal OVA challenge. In rat models, the most common sensitization regimen is 0.3 mg OVA combined with 30 mg aluminum hydroxide in 1 mL saline, administered intraperitoneally every other day for 7 doses; local challenge uses 5 mg OVA (∼50 µL of 10% OVA solution), with observation typically performed 30 min post-challenge (18). Some studies have shown that three intraperitoneal injections (on days 1, 3, and 7) are sufficient to induce typical and pronounced AR inflammatory responses (19). In mouse models, the most common sensitization dose is 50 µg OVA with 1 mg Al(OH)₃ in 200 µL, administered once weekly for 3 weeks; local challenge uses 500 µg OVA (∼20 µL of 2.5% OVA solution) intranasally once daily for 6 days (20).

With advancing understanding of AR mechanisms, the classical OVA model no longer fully meets research demands for chronic inflammation, airway remodeling, or disease-syndrome integration. Multiple modifications have therefore been introduced: (1) Extended challenge duration—prolonging intranasal challenge from 7 days to 4–12 weeks to establish chronic AR models, leading to persistent Th2 inflammation, goblet cell hyperplasia, mucus hypersecretion, and basement membrane thickening, more closely mirroring persistent AR pathology (21); (2) Alternative sensitization routes—including subcutaneous injection, intranasal instillation, nebulized inhalation, and epicutaneous sensitization. Intranasal sensitization better resembles natural inhalation, reducing systemic immune bias, while nebulized inhalation simulates continuous airborne allergen exposure, enhancing clinical relevance; (3) Combined adjuvants or co-stimuli—some studies co-administer PT, LPS, or TCM syndrome-inducing factors (e.g., tobacco smoke, cold stress, high-fat diet, bitter-cold purgatives) to construct disease-syndrome integrated models for herbal efficacy evaluation and syndrome mechanism research.

The widespread use of OVA is mainly attributable to its excellent experimental controllability. Compared with natural aeroallergens, OVA possesses highly stable physicochemical properties, low batch-to-batch variability, and mature commercial preparations, resulting in excellent inter-laboratory reproducibility. Moreover, sensitization protocols have been extensively optimized over the past three decades with respect to allergen dose, challenge frequency, sampling time, and outcome evaluation, making OVA particularly suitable for mechanistic studies and pharmacological efficacy assessments (11).

Nevertheless, OVA is not a naturally occurring inhaled allergen in humans. Unlike house dust mite allergens, OVA lacks intrinsic protease activity and therefore cannot directly disrupt epithelial tight junctions or activate epithelial-derived alarmins, including IL-25, IL-33, and thymic stromal lymphopoietin (TSLP). Consequently, OVA-induced inflammation relies predominantly on artificial immune sensitization with aluminum hydroxide, whereas HDM-induced models more faithfully reproduce epithelial barrier dysfunction and innate immune activation observed in patients with allergic rhinitis (15, 17). This limitation should be considered when selecting models for studies focusing on epithelial immunity or early allergic sensitization.

In recent years, the classical OVA model has been continuously optimized to improve its translational relevance. For example, combined OVA/protease-induced models have been developed to better mimic the protease-rich microenvironment of natural aeroallergens and to enhance epithelial barrier injury and type 2 inflammation. These modified models produce more pronounced eosinophilic infiltration, mucus hypersecretion, and histopathological changes than conventional OVA models and are increasingly employed in pharmacological efficacy studies. In parallel, OVA-based models have become the preferred platform for evaluating natural products and novel therapeutic interventions. Recent experimental studies have demonstrated that omega-3 fish oil and Nigella sativa oil effectively attenuate nasal inflammation, reduce eosinophil infiltration, suppress Th2 cytokine production, and improve histopathological injury in OVA-induced AR models, highlighting the continued value of standardized OVA models for preclinical drug screening and mechanistic investigations (22, 23).

#### 3.6.2 HDM-Based modeling

No standardized protocol currently exists for HDM models, with substantial variation in sensitization methods, dosages, and timelines across studies. HDM extracts contain multiple natural allergens, including Der p1 and Der p2, along with protease components that serve as natural adjuvants. Adjuvants such as aluminum hydroxide are generally not required; subcutaneous sensitization combined with intranasal challenge, or direct repeated intranasal instillation, is commonly used. Compared with OVA models, HDM models more closely mimic natural inhalation sensitization and offer higher clinical relevance, particularly for studying epithelial barrier damage, innate immune activation, and chronic inflammatory mechanisms. However, batch-to-batch variability in HDM extracts from different manufacturers reduces reproducibility, and standardization remains to be improved (24).

#### 3.6.3 Pollen-Based modeling

Pollen is the most important natural sensitizing allergen for seasonal AR, including Japanese cedar pollen, ragweed pollen, mugwort pollen, and birch pollen. Pollen models typically use subcutaneous sensitization followed by intranasal challenge with pollen extracts, or direct repeated intranasal instillation. Compared with OVA models, regional differences in pollen species and allergen composition, as well as variability in extract purity and activity influenced by collection season and storage conditions, result in relatively lower reproducibility. These models are predominantly used for seasonal AR and environmental factor research (25).

#### 3.6.4 TDI-Based modeling

TDI is primarily used for modeling occupational AR and occupational asthma. TDI is not a protein antigen itself but acts as a hapten, binding to host proteins to form complete antigens that trigger immune responses. Repeated intranasal instillation or topical application is typically used to induce sneezing, rhinorrhea, nasal itching, and nasal mucosal inflammation. TDI models have the advantages of short induction periods and good reproducibility; however, their volatility and toxicity can cause significant neurogenic inflammation and local tissue damage, posing risks to both animals and personnel. Consequently, TDI models are mainly confined to occupational allergy and environmental toxicology research and are less commonly used for TCM syndrome modeling (26).

### 3.7 TCM syndrome induction methods

Currently, the most frequently modeled TCM syndromes in AR animal studies are Lung Qi Deficiency, Spleen Qi Deficiency, and Kidney Yang Deficiency, with well-established protocols for each. To enhance clinical relevance, increasing research has focused on compound syndrome models, including “Lung-Spleen Qi Deficiency,” “Spleen Deficiency with Dampness Retention,” “Cold-Heat Complex,” “Lung-Kidney Yang Deficiency,” and “Spleen-Kidney Yang Deficiency.” All TCM syndrome models are established on the basis of classical AR disease models; therefore, OVA sensitization details are not repeated in this section.

#### 3.7.1 Lung Qi deficiency (cold) pattern

The Lung Qi Deficiency-Cold pattern accounted for the highest proportion (34.69%) among TCM syndromes. The modeling approach follows the TCM theory that “cold exposure and cold beverage intake impair the lungs.” Most studies refer to the simple tobacco smoke exposure method described in Medical Animal Experiment Methods and Practical TCM Syndrome Animal Model Science. This method involves burning specially formulated moxa sticks (containing sulfur and mugwort) or wood shavings/sawdust to generate smoke, which is used to fumigate rats for 14 consecutive days to induce Qi depletion and impaired lung function. Cold stimulation (e.g., ice-water swimming or cold air exposure) is often added to exacerbate cold manifestations (27). Model success is characterized by sneezing, nasal rubbing, and rhinorrhea, accompanied by cough weakness, tachypnea, lethargy, dull fur, and reduced weight gain, consistent with the Lung Qi Deficiency-Cold pattern.

Studies have shown that chronic tobacco smoke exposure suppresses pulmonary T-cell IFN-γ secretion and affects dendritic cell-mediated T-cell polarization, leading to Th1/Th2 imbalance and promoting Th2-type inflammation (28). This suggests that prolonged smoke and cold stimulation may not only induce chronic airway inflammation and oxidative stress but also amplify OVA-induced Th2 skewing by attenuating Th1 immune responses, thereby generating immunological features more closely resembling “Lung Qi insufficiency with weakened superficial defense.”

#### 3.7.2 Spleen Qi deficiency pattern

Spleen Qi Deficiency modeling employs diverse approaches, commonly using single-factor or multi-factor composite methods. Single-factor models primarily use bitter-cold purgative agents such as rhubarb cold-soak extract or senna leaf decoction by gavage to deplete spleen Qi (29). Multi-factor composite models often combine irregular diet with physical exhaustion, such as exhaustive swimming or intermittent fasting (30). Successful models exhibit AR nasal symptoms along with typical Spleen Qi Deficiency manifestations, including reduced food intake, abdominal distension, loose stools, fatigue, and slow weight gain—with nasal symptoms and inflammatory responses generally more severe than in AR-only models (31).

Recent studies have proposed that Spleen Qi Deficiency may be closely associated with gut microbiota dysbiosis and intestinal barrier dysfunction. Spleen deficiency-induced disruption of intestinal microecological homeostasis can increase intestinal permeability and promote chronic low-grade inflammation, subsequently affecting respiratory immune regulation via the “gut-lung axis.” Evidence indicates that microbial imbalance can promote Treg/Th17 immune imbalance and enhance Th2-type inflammation, thereby aggravating allergic airway disease (32–34). The immunological basis of Spleen Qi Deficiency may therefore extend beyond digestive dysfunction to encompass systemic immune homeostasis dysregulation and mucosal immune abnormalities.

#### 3.7.3 Kidney yang deficiency pattern

Kidney Yang Deficiency models are primarily induced by pharmacological agents, including glucocorticoid administration, adenine gavage, and hydroxyurea. Glucocorticoid injection is a common method, but its inherent anti-inflammatory and immunosuppressive properties may confound AR experimental results. Consequently, adenine gavage has been explored and validated as a preferable alternative. Adenine induces damage to renal tubules and testicular spermatogenic epithelium, simulating the state of “kidney governing reproduction” insufficiency, with manifestations including cold intolerance, curled posture, and rough fur (35). Some studies have reported that hydroxyurea achieves shorter modeling duration, better sensitization, and greater stability with lower mortality compared to glucocorticoid or adenine-based models (36, 37).

It is well established that Kidney Yang Deficiency induction methods—including adenine, hydroxyurea, and prolonged glucocorticoid intervention—affect hypothalamic-pituitary-target gland axis function, particularly the hypothalamic-pituitary-adrenal (HPA) axis and hypothalamic-pituitary-gonadal (HPG) axis, resulting in reduced body temperature, decreased activity, and dull fur. Relevant studies have found that Kidney Yang Deficiency AR models frequently exhibit decreased IFN-*γ*, elevated IL-4, and T-bet/GATA-3 imbalance, suggesting involvement of neuro-endocrine-immune network dysregulation (38). Dysregulation of glucocorticoid signaling due to HPA axis hypofunction may represent a key mechanism underlying Th1/Th2 and Th17/Treg immune imbalance in the Kidney Yang Deficiency state (39).

#### 3.7.4 Other syndrome types and compound syndromes

Currently, deficiency patterns (Lung Qi Deficiency-Cold, Spleen Qi Deficiency, Kidney Yang Deficiency) dominate TCM syndrome models for AR, with proportions far exceeding excess patterns. This may reflect the inherent difficulty in modeling dynamic excess states through simple external stimulation.

For the “Lung Meridian Latent Heat” pattern, some researchers have used a compound hot-nature herbal formula (prepared Aconite, cinnamon bark, dried ginger) by gavage to induce heat-pattern guinea pig models. Successful modeling is indicated by hyperphagia, increased water intake, hyperactivity, loud vocalization, irritability, difficult defecation with dry hard dark-colored stool, aggressive behavior, normal fur, and reddish tongue (40).

The “Cold-Heat Complex” pattern can be modeled using a multi-factor stratification approach: propylthiouracil to inhibit thyroid function, ice-water cold stress, and prolonged bitter-cold herbal administration to deplete Yang Qi (creating a Yang deficiency-cold foundation), combined with gypsum-anemarrhena decoction to clear stomach heat, senna leaf to drain intestinal heat, and subcutaneous dried yeast to induce systemic fever (creating excess heat manifestations). Rats simultaneously exhibit cold intolerance (deficiency-cold) and fever, thirst, and constipation (heat signs), forming a cold-heat complex model (41).

Other compound syndromes combine two single-syndrome approaches. “Lung-Spleen Qi Deficiency” uses tobacco smoke exposure + rhubarb decoction gavage + dietary control (42). “Spleen Deficiency with Dampness Retention” uses senna leaf gavage + lard gavage + alternate-day fasting (30). “Lung-Kidney Yang Deficiency” combines tobacco smoke exposure + cold stimulation + hydrocortisone injection (43). “Spleen-Kidney Yang Deficiency” combines hydrocortisone injection + rhubarb decoction gavage (44).

### 3.8 Observation and evaluation indicators

Evaluation criteria for AR models, including disease-syndrome integrated models, typically combine subjective and objective indicators. The primary evaluation standard is behavioral scoring, with the vast majority of studies adopting the scoring system established by Zhao et al. (45), which scores the frequency and severity of nasal rubbing, sneezing, and rhinorrhea over a defined observation period (10–30 min). A total score >5 is considered indicative of successful AR modeling (46). TCM syndrome model evaluation is predominantly subjective: the appearance of external syndrome manifestations corresponding to the modeled pattern in experimental animals is considered evidence of successful modeling.

Objective evaluation indicators are more diverse and include serum immunological markers (OVA-specific IgE, total IgE, IL-4, IL-5, IL-13, IFN-*γ*, etc.) (47), passive cutaneous anaphylaxis tests, nasal lavage fluid analysis (eosinophil counts), and nasal mucosal histopathological examination (eosinophil and mast cell infiltration, epithelial damage, goblet cell hyperplasia, submucosal gland hyperplasia, and vasodilation) (48, 49).

### 3.9 Immunological mechanisms in TCM syndrome AR models

Advances in immunology have extended the understanding of AR pathogenesis beyond classical IgE-mediated type I hypersensitivity to a broader type 2 inflammation-centered immune regulatory network. Allergens first act on nasal epithelial cells, inducing epithelial-derived alarmins such as TSLP, IL-25, and IL-33, which activate ILC2s and Th2 cells, promoting secretion of IL-4, IL-5, and IL-13, ultimately leading to eosinophil recruitment, B-cell IgE class switching, and persistent allergic inflammation (50–52). AR is now considered a type 2 inflammatory disease involving both innate and adaptive immunity.

In AR animal models, Th1/Th2 immune imbalance remains a key indicator for evaluating model stability and therapeutic efficacy. Yu et al. demonstrated that serum IL-4 levels were significantly elevated while IFN-γ expression decreased in AR model rats, indicating Th2-skewed immune responses; TCM intervention reduced IL-4 and increased IFN-γ, suggesting that TCM may exert anti-inflammatory and immunomodulatory effects by restoring Th1/Th2 balance (47).

However, the classical Th1/Th2 paradigm alone is insufficient to fully explain the complex immunopathology of AR. Treg/Th17 imbalance has been identified as a critical mechanism in AR chronicity. Treg cells maintain immune tolerance through IL-10 and TGF-β secretion, while Th17 cells promote tissue inflammation via IL-17A and IL-22. Studies have found that Treg cell proportions are decreased while Th17 cells are increased in peripheral blood and nasal mucosa of AR patients, with the degree of imbalance correlating with disease severity (33).

Beyond immune cell abnormalities, nasal epithelial barrier damage has recently become a major research focus. Tight junction proteins such as ZO-1, Occludin, and Claudin maintain mucosal integrity under normal conditions, preventing allergen penetration into submucosal tissues. Studies have shown significantly reduced ZO-1 and Occludin expression in AR nasal mucosa, with increased epithelial permeability facilitating sustained allergen penetration and amplified local inflammation (53). This suggests that AR is not only an immune imbalance disease but also a mucosal barrier dysfunction disease.

Evidence from TCM syndrome models indicates that different TCM syndromes may correspond to distinct immune-inflammatory states. The Lung Qi Deficiency-Cold pattern is commonly characterized by elevated IL-4 and IgE with decreased IFN-*γ*, suggesting persistent Th2 skewing and impaired mucosal immune defense. The Spleen Qi Deficiency pattern is often accompanied by gut microbiota dysbiosis, impaired intestinal barrier function, and chronic low-grade inflammation, potentially affecting respiratory immune homeostasis through the gut-lung axis. The Kidney Yang Deficiency pattern frequently manifests as hypothalamic-pituitary-target gland axis dysfunction and neuro-endocrine-immune network imbalance, suggesting immune hyporesponsiveness and impaired inflammatory regulation (39).

With the development of systems biology technologies, multi-omics approaches have become important tools for exploring the nature of TCM syndromes. Transcriptomic studies have revealed significant differences in cytokine signaling pathways, oxidative stress, and energy metabolism-related gene expression across different AR syndrome models. Metabolomic analyses have shown that abnormalities in amino acid, lipid, and energy metabolism may participate in the formation of Lung-Spleen Qi Deficiency and Kidney Yang Deficiency patterns. Gut microbiome sequencing has revealed that AR models commonly exhibit reductions in beneficial bacteria such as Lactobacillus and Bifidobacterium with increases in opportunistic pathogens, closely correlated with Th17/Treg imbalance and type 2 inflammation severity (54).

### 3.10 Animal welfare considerations and ethical implications

Animal welfare is an important but often under-discussed aspect of allergic rhinitis (AR) syndrome-integrated animal modeling. In addition to allergen sensitization and challenge procedures, many traditional Chinese medicine (TCM) syndrome-induction protocols involve cold exposure, smoke stimulation, exhaustive swimming, fasting, sleep disturbance, irritant gavage, or drug-induced deficiency states, all of which may impose substantial physiological and psychological stress on animals. Although these procedures are intended to mimic syndrome-specific pathophysiological states such as Lung Qi Deficiency, Spleen Qi Deficiency, Kidney Yang Deficiency, or heat-related syndromes, they may also activate non-specific stress–neuroendocrine–immune pathways, thereby affecting inflammatory outcomes, immune-cell polarization, and behavioral phenotypes independent of AR itself (38, 39).

From a mechanistic perspective, stress-related interventions may influence the hypothalamic–pituitary–adrenal (HPA) axis, sympathetic nervous system activity, glucocorticoid secretion, and neuroimmune crosstalk, which in turn modulate Th1/Th2/Th17/Treg balance, epithelial barrier function, and systemic inflammatory responses (39). Therefore, some observed “syndrome phenotypes” may partly reflect generalized stress responses rather than syndrome-specific biological features. This issue is particularly relevant for syndrome-induction methods based on chronic cold exposure, repeated forced swimming, prolonged food restriction, or tobacco-smoke exposure, as these procedures may introduce confounding effects on immune and metabolic readouts. For example, cigarette smoke exposure has been shown to suppress pulmonary T-cell responses and alter host immune defense in murine models, highlighting the possibility that smoke-based syndrome induction may affect AR-related immune outcomes beyond the intended syndrome simulation (28).

Accordingly, the design of AR–TCM syndrome animal models should place greater emphasis on animal welfare, severity control, and protocol refinement. First, the scientific necessity of high-burden interventions should be explicitly justified, particularly when multiple stressors are combined with allergen sensitization. Second, humane endpoints should be prospectively defined and consistently monitored, including excessive body-weight loss, dehydration, persistent hypoactivity, abnormal posture, severe piloerection, self-injury, respiratory distress, or inability to access food and water. Third, investigators should clearly report whether any analgesic, supportive, or early-withdrawal measures were used when animals developed severe stress reactions or marked model-related morbidity.

More broadly, future studies should align AR syndrome-model design with the 3Rs principle (Replacement, Reduction, and Refinement) and with contemporary reporting recommendations such as ARRIVE 2.0 (55). Refinement is particularly important in this field, because many TCM syndrome models rely on chronic environmental or pharmacological stressors that may be difficult to standardize and may cause unnecessary suffering if not carefully controlled. Whenever possible, lower-burden and better-standardized procedures should be prioritized, and syndrome induction should be calibrated to the minimum intensity necessary to reproduce stable phenotypes. In addition, animal welfare information—including mortality, attrition, signs of distress, and reasons for exclusion—should be transparently reported, as these factors directly influence both ethical acceptability and model reproducibility.

Taken together, animal welfare is not only an ethical requirement but also a methodological issue that directly affects the reliability and translational value of AR syndrome-integrated animal models. Future studies should therefore move beyond simply describing syndrome-induction procedures and instead provide a more balanced evaluation of model validity, reproducibility, and welfare burden, thereby improving both the scientific rigor and ethical quality of this research field.

## 4 Discussion: challenges and future directions in AR disease-syndrome integrated animal models

One notable finding of this review is that disease–syndrome integrated TCM animal models currently represent only a small proportion of the published literature. Among the 470 included studies, only 49 established explicit TCM syndrome models, whereas the majority focused on conventional AR animal models. Consequently, the present review first summarizes the methodological basis of conventional AR models before discussing advances in disease–syndrome integrated modeling. This structure reflects the current state of evidence rather than an equal distribution of available studies.

First, the majority of current models employ artificial antigens such as OVA, which differ from the natural sensitization process in humans. OVA requires intraperitoneal injection with exogenous adjuvants, limiting clinical translatability. Greater emphasis should be placed on natural allergen models, particularly HDM and pollen, to improve model authenticity and clinical relevance.

Second, TCM syndrome evaluation relies on “four-examination integration” (sizhen hecan), but current assessment indicators are predominantly subjective qualitative or semi-quantitative measures. The lack of objective syndrome-reflective biomarkers compromises comparability across studies. Future research should focus on establishing an objective, reproducible evaluation system that integrates behavioral scoring with immunological, biochemical, and histopathological parameters, enhancing the objectivity and international acceptance of TCM syndrome models.

Third, non-deficiency patterns such as Lung Meridian Latent Heat and Cold-Heat Complex remain understudied, failing to cover the full spectrum of clinical AR syndrome differentiation. Expanding research on excess and complex syndrome patterns is essential to comprehensively represent clinical diversity.

Fourth, TCM syndrome induction typically requires superimposing multiple stressors—cold exposure, tobacco smoke, exhaustive swimming, fasting, and pharmacological interventions—which may induce sustained stress, pain, myelosuppression, gastrointestinal injury, or hepatic/renal dysfunction, potentially confounding behavioral and inflammatory assessments. Optimizing modeling protocols to reduce stimulus intensity and duration is necessary.

Looking forward, several directions merit priority:(1)Standardization of disease–syndrome integrated modeling:Future studies should establish a standardized framework for allergic rhinitis (AR)–traditional Chinese medicine (TCM) syndrome integrated animal modeling, including unified descriptions of animal species and strains, sensitization protocols, syndrome-induction procedures, and criteria for successful model establishment. Such standardization would improve inter-study comparability, methodological reproducibility, and translational interpretability, thereby providing a more robust experimental basis for mechanistic studies and therapeutic evaluation in AR syndrome models.(2)Strengthening objective evaluation of TCM syndrome models:The evaluation of TCM syndrome models should not rely solely on subjective external manifestations such as lethargy, fur appearance, appetite, stool changes, or activity level. Instead, future studies should incorporate objective and reproducible multidimensional indicators, including total IgE and allergen-specific IgE, Th1/Th2/Th17/Treg-related cytokines, eosinophil infiltration and nasal histopathology, body-weight changes and metabolic parameters, microbiome- and multi-omics-based biomarkers, as well as blinded behavioral scoring systems. Such a multidimensional evaluation framework would improve the objectivity, reproducibility, and biological interpretability of syndrome-model assessment.(3)Improving methodological rigor in animal experiments:Future AR animal studies should further strengthen methodological rigor by providing clearer reporting of randomization procedures, blinding implementation, sample-size estimation, animal attrition and model failure, predefined outcome measures, and strategies for controlling selective reporting. Improved methodological transparency will not only reduce the risk of bias but also enhance the credibility and translational value of preclinical evidence.(4)Enhancing translational validity:Greater emphasis should be placed on the translational relevance of AR–TCM syndrome animal models to human AR phenotypes and endotypes, including their correspondence with airway inflammatory patterns, neuroendocrine–immune regulatory networks, and clinically recognized patient subgroups. Future work should aim to clarify whether syndrome-integrated animal models can better reflect the heterogeneity of human AR and thereby provide a more meaningful platform for mechanism exploration and therapeutic evaluation.(5)Strengthening animal welfare and refinement:Modeling procedures involving smoke exposure, cold stimulation, forced swimming, fasting, or toxic stimulation should be critically justified and carefully refined to minimize unnecessary animal distress while preserving model validity. Future studies should more explicitly incorporate the principles of Replacement, Reduction, and Refinement (3Rs), establish humane endpoints, and provide a transparent justification of model severity. Strengthening animal-welfare considerations will be essential for improving both the ethical quality and scientific robustness of AR syndrome-model research.

## 5 Conclusion

This systematic review comprehensively summarizes the current landscape of animal models of allergic rhinitis (AR) and disease–syndrome integrated models based on Traditional Chinese Medicine (TCM) syndromes. Although conventional AR animal models currently constitute the majority of available evidence, disease–syndrome integrated TCM models are becoming an increasingly important research direction and are expected to play a greater role in mechanistic studies and translational TCM research.

Future research should prioritize natural allergen-based models, develop multidimensional evaluation systems integrating “symptom-immunology-omics” approaches, and adhere to rigorous methodological standards and ethical principles. These efforts will provide a more reliable experimental foundation for elucidating TCM mechanisms and advancing personalized, syndrome-based therapeutic strategies for AR.

## Data Availability

The original contributions presented in the study are included in the article/Supplementary Material, further inquiries can be directed to the corresponding author.
